# Health Behavior of Adults Without Cognitive Impairment After Receiving Amyloid-β PET Results

**DOI:** 10.1001/jamanetworkopen.2025.45774

**Published:** 2025-12-01

**Authors:** Lindsay R. Clark, Claire M. Erickson, Kristin E. Basche, Fred B. Ketchum, Amanda J. Peterson, Hannah L. Rosario, Nathaniel A. Chin

**Affiliations:** 1 Division of Geriatrics and Gerontology, Department of Medicine, University of Wisconsin School of Medicine and Public Health, Madison; 2 Geriatric Research Education and Clinical Center, William S. Middleton Memorial Veterans Hospital, Madison, Wisconsin; 3 Banner Alzheimer’s Institute, Phoenix, Arizona; 4 Department of Neurology, University of Wisconsin School of Medicine and Public Health, Madison

## Abstract

This cohort study explores self-reported health behavior changes of adults without cognitive impairment after receiving amyloid-β positron emission tomography (PET) results.

## Introduction

Biomarker testing may be key to the timely diagnosis of Alzheimer disease, as it allows for detection of pathophysiologic changes before symptom onset. Prior studies have shown that cognitively unimpaired adults screened for psychological readiness understand their biomarker results and experience minimal postdisclosure psychological effects.^1,2,3^ This study evaluated whether adults without cognitive impairment reported health behavior change after receiving amyloid-β (Aβ) results.

## Methods

The University of Wisconsin–Madison Institutional Review Board approved this cohort study. Older adults (aged 65-82 years) recruited from the Wisconsin Registry for Alzheimer Prevention (WRAP) provided signed informed consent. Data were collected between October 2020 and February 2023.^4^ We followed the STROBE reporting guideline.

Participants received a positron emission tomography (PET) result indicating elevated (positive) or nonelevated (negative) Aβ and completed a dementia risk-reduction counseling visit. Clinicians reviewed standard brain health recommendations, discussed participants’ medical history and risk factors, and facilitated development of a brain health–focused goal. Brief validated self-report measures of physical activity, cognitive activity, social activity, diet, sleep quality, and stress reduction activity were administered before and 7 months after disclosure (eTable in Supplement 1). The primary outcome was predisclosure to postdisclosure health behavior change, evaluated with the paired *t* or Wilcoxon signed-ranked test (*V*). Changes by amyloid result were compared using analysis of covariance. Brain health goal progress was also assessed. Analyses were conducted using R, version 4.4.3 (R Project for Statistical Computing), from January 1 to June 30, 2025. Two-sided α = .05 was significant.

## Results

Ninety-nine adults enrolled (mean [SD] age, 72.0 [4.8] years; 66 females [67.7%] and 33 males [33.3%]) and received Aβ results (Aβ-positive, 28 [28.3%]; Aβ-negative, 71 [71.7%]). Race and ethnicity was self-reported as Asian (1 [1.0%]), Black or African American (4 [4.0%]), or non-Hispanic White (94 [94.9%]). Most participants (97 [98.0%]) completed follow-up.

Participants endorsed greater likelihood to make lifestyle changes to improve brain health at follow-up compared with baseline (*t*_93_ = −2.42; *P* = .02). However, no predisclosure to postdisclosure change in self-reported physical activity (*V* = 2005; *P* = .39), diet (*t*_92_ = 1.69; *P* = .09), social activity (*t*_92_ = 1.15; *P* = .25), stress reduction activity (*V* = 316; *P* = .75), or sleep quality (*V* = 881; *P* = .49) was observed (Figure). Significantly less cognitive activity was reported at follow-up (*t*_93_ = 3.92; *P* < .001).

**Figure.**
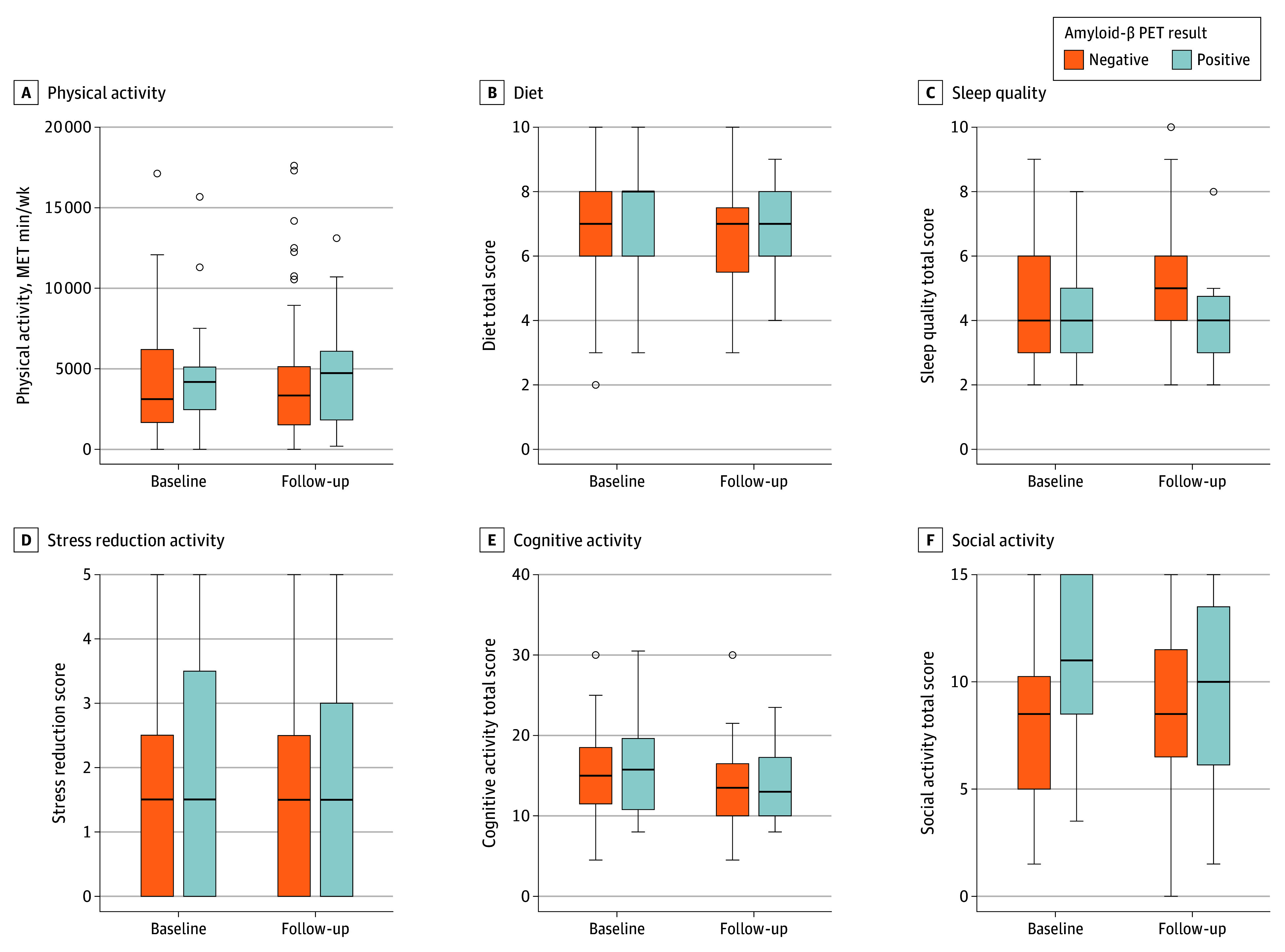
Self-Reported Health Behaviors by Amyloid-β Positron Emission Tomography (PET) Groups Changes in self-reported health behaviors (eTable in Supplement 1) at baseline and at 7-month follow-up in older adults with elevated (positive) and nonelevated (negative) amyloid-β PET results. Horizontal lines and boxes represent medians and IQRs; error bars represent 95% CIs. A, Physical activity is reported in metabolic equivalent of task (MET) minutes of time spent walking or in moderate or vigorous activity per week. B, Diet total score is the frequency of fruit and vegetable consumption in the past month. C, Sleep quality total score is the frequency of restful sleep and trouble sleeping in the past month. D-F, Stress reduction, cognitive, and social activity total scores are hours spent during a typical week in the past month.

No follow-up differences between Aβ groups were observed for physical activity, diet, social activity, cognitive activity, stress reduction activity, or likelihood to make behavior changes (Table). The Aβ-negative group reported better mean (SD) sleep quality scores at follow-up (4.84 [1.8]) than the Aβ-positive group (3.92 [1.6]) (*P* = .03). Most participants (83 [85.6%]) reported progress on individual brain health goals; 47 (48.5%) met or exceeded their goal and 36 (37.5%) reported some progress (13 [13.5%] reported no progress). No difference in goal progress was observed between Aβ groups (χ^2^_2_ = 0.87; *P* = .65).

**Table.** Health Behavior Change After Receiving Amyloid-β Results^a^

Parameter	Health behavior											
	Physical activity		Diet		Sleep quality		Cognitive activity		Social activity		Stress reduction activity	
	*F*_1,88_	*P* value	*F*_1,87_	*P* value	*F*_1,89_	*P* value	*F*_1,89_	*P* value	*F*_1,87_	*P* value	*F*_1,87_	*P* value
Age	0.03	.86	5.03	.03	0.09	.76	3.67	.06	1.51	.22	0.07	.80
Education	0.95	.33	5.66	.02	0.65	.42	1.92	.17	0.51	.48	0.87	.35
Sex												
Female	1.56	.22	2.23	.14	8.86	<.01	2.46	.12	0.26	.61	6.40	.01
Male	Reference	NA	Reference	NA	Reference	NA	Reference	NA	Reference	NA	Reference	NA
Baseline report	13.88	<.001	27.85	<.001	79.81	<.001	32.62	<.001	55.06	<.001	90.79	<.001
Amyloid-β PET result												
Positive (elevated)	0.01	.91	0.46	.50	4.88	.03	0.14	.71	2.50	.12	1.62	.59
Negative (nonelevated)	Reference	NA	Reference	NA	Reference	NA	Reference	NA	Reference	NA	Reference	NA

Abbreviations: NA, not applicable; PET, positron emission tomography.

^a^ Calculated using analysis of covariance models.

## Discussion

Most participants reported progress on brain health goals, and the overall sample expressed greater interest in brain health behavior at follow-up. However, participants did not exhibit significantly different activity levels from baseline on self-report scales. The Aβ-positive group also did not report significantly increased activity levels. The discrepancy between earlier descriptive reports of increased behavior changes after receiving elevated amyloid PET results^5^ and the current findings suggest personalized health behavior measures that vary across individuals may be necessary to adequately capture progress. Alternatively, people may desire dementia risk reduction but encounter barriers to health behavior changes. The WRAP promotes brain health; thus, participants may have engaged in health behaviors before receiving their PET result. Previous findings demonstrated that health behavior change with sedentary at-risk adults is both feasible and effective in reducing cognitive decline.^6^

Study limitations include potential bias in available measures and limited generalizability due to sample bias. Larger studies are needed to understand whether and how the general population acts on dementia risk information and to identify targets to inform personalized risk-reduction interventions.

## Notes

Supplement 1. **eTable.** Health Behavior Measures

Supplement 2.
Data Sharing Statement

## References

[1] Grill JD, Raman R, Ernstrom K, ; A4 Study Team. Short-term psychological outcomes of disclosing amyloid imaging results to research participants who do not have cognitive impairment. JAMA Neurol. 2020;77(12):1504-1513. doi:10.1001/jamaneurol.2020.2734 32777010 PMC7418046

[2] Clark LR, Erickson CM, Chin NA, . Psychosocial implications of learning amyloid PET results in an observational cohort. Alzheimers Dement. 2024;20(9):6579-6589. doi:10.1002/alz.14153 39129396 PMC11497643

[3] van der Schaar J, Visser LNC, Ket JCF, . Impact of sharing Alzheimer’s disease biomarkers with individuals without dementia: a systematic review and meta-analysis of empirical data. Alzheimers Dement. 2023;19(12):5773-5794. doi:10.1002/alz.13410 37496313

[4] Erickson CM, Chin NA, Rosario HL, Peterson A, Johnson SC, Clark LR. Feasibility of virtual Alzheimer’s biomarker disclosure: findings from an observational cohort. Alzheimers Dement (N Y). 2023;9(3):e12413. doi:10.1002/trc2.12413 37521522 PMC10382796

[5] Largent EA, Harkins K, van Dyck CH, Hachey S, Sankar P, Karlawish J. Cognitively unimpaired adults’ reactions to disclosure of amyloid PET scan results. PLoS One. 2020;15(2):e0229137. doi:10.1371/journal.pone.0229137 32053667 PMC7018056

[6] Baker LD, Espeland MA, Whitmer RA, . Structured vs self-guided multidomain lifestyle interventions for global cognitive function: the US POINTER randomized clinical trial. JAMA. 2025;334(8):681-691. doi:10.1001/jama.2025.12923 40720610 PMC12305445

