# Supplementary material for: Health Behavior of Adults Without Cognitive Impairment After Receiving Amyloid-β PET Results
Source: JAMA Netw Open. 2025 Dec 1;8(12):e2545774. doi: 10.1001/jamanetworkopen.2025.45774 (PMC12670200; doi:10.1001/jamanetworkopen.2025.45774)
Supplement: Supplement 1. — eTable. Health Behavior Measures [file jamanetwopen-e2545774-s001.pdf]

Supplemental Online Content

Clark LR, Erickson CM, Basche KE, et al. Health behavior of adults without cognitive impairment after receiving amyloid-β PET results. *JAMA Netw Open*. 2025;8(12):e2545774. doi:10.1001/jamanetworkopen.2025.45774

eTable. Health Behavior Measures

This supplemental material has been provided by the authors to give readers additional information about their work.

© 2025 Clark LR et al. *JAMA Network Open*.

eTable. Health Behavior Measures

Behavior	Measure, Number Items, Response Type	Outcome variable
**Physical activity**	The International Physical Activity Questionnaire – Short Form (IPAQ) includes items that estimate total physical activity from time spent walking or in moderate or vigorous activity in metabolic equivalent (MET)-min/week. Each item response was converted to total minutes per day.	MET-min/week was calculated for each category using published criteria for walking (3.3*minutes*days), moderate activity (4.0*minutes*days), and vigorous activity (8.0*minutes*days). The total combined MET-min/week were summed (METS_walking + METS_moderate + METS vigorous).
**Cognitive activity**	Subset of items from the Community Health Activities Model Program for Seniors (CHAMPS) questionnaire. A 4-item Likert scale indicated the number hours spent in each activity during a typical week over the last month (0 hours, 1-2 hours, 2-5 hours, or >5 hours). Items included: 1) Doing woodworking, needlework, drawing, or other arts and crafts, 2) Attending a movie, lecture, or sport event, 3) Puzzling, playing cards, bingo, board or computer games, 4) Reading (book, newspaper, Kindle, etc), 5) Playing a musical instrument, 6) Working on your car, truck, lawn mower, or other machinery, and 7) Listening to your favorite music, podcast, or audio book.	Responses were coded such that 0 hours=0, 1-2 hours=1.5, 2-5 hours=3.5, and >5 hours=5. Responses for the seven items were summed, and the total score was used as the outcome variable in analyses.
**Social activity**	Subset of items from the Community Health Activities Model Program for Seniors (CHAMPS) questionnaire. A 4-item Likert scale indicated the number hours spent in each activity during a typical week over the last month (0 hours, 1-2 hours, 2-5 hours, or >5 hours). Items included: 1) Visiting with friends or family, 2) Attending a group or club activity (i.e. church, book club, rotary, etc), and 3) Employment or volunteer-related activities with other people (i.e. at a food bank or school).	Responses were coded such that 0 hours=0, 1-2 hours=1.5, 2-5 hours=3.5, and >5 hours=5. Responses for the three items were summed, and the total score was used as the outcome variable in analyses.
**Stress reduction activity**	One item from the Community Health Activities Model Program for Seniors (CHAMPS) questionnaire was included: 1) Meditating, mindfully breathing, doing yoga, stretching, or massage. A 4-item Likert scale indicated the number hours spent in each activity during a typical week over the last month (0 hours, 1-2 hours, 2-5 hours, or >5 hours).	Responses were coded such that 0 hours=0, 1-2 hours=1.5, 2-5 hours=3.5, and >5 hours=5.
**Sleep quality**	Two items from the Medical Outcomes Study (MOS) Sleep Scale were included: 1) How often during the past month did you get enough sleep to feel rested upon waking in the morning? and 2) How often during the past month did you have trouble falling asleep?. Participants responded to a 6-item scale indicating frequency (all of the time [1], most of the time [2], a	Responses to the first item were reverse coded, such that for both items a higher score equated to better sleep quality. Responses were summed and the total score was used as the outcome variable in analyses.

© 2025 Clark LR et al. *JAMA Network Open*.

	good bit of the time [3], some of the time [4], a little of the time [5], and none of the time [6]).	
**Diet**	Two items were included: “Over the last month, how often have you eaten vegetables (like lettuce, spinach, cucumber, sweet potato)?” and “Over the last month, how often have you eaten fruit?”. 5-item Likert scale indicating frequency of consumption (rarely [1], sometimes [2], often [3], usually [4], every meal [5]).	Responses were summed and the total score was used as the outcome variable in analyses.
**Health behavior change likelihood**	Participants were prompted to “Think about making changes in your life, how likely are you to change your behavior in the following activities as a way to improve brain health” and respond to the following 6 items: “Eating a healthier diet,” “Increasing your exercise,” “Getting enough sleep,” “Reducing stress,” “Doing more brain games,” and “Seeing more of your friends or family.” Scale from 0 to 10 with 0 indicating Not Likely, 5 indicating Somewhat Likely and 10 indicating Very Likely.	Responses to the six items were averaged and the average score was used as the outcome variable in analyses.

© 2025 Clark LR et al. *JAMA Network Open*.
