# Supplementary material for: Health Behavior of Adults Without Cognitive Impairment After Receiving Amyloid-β PET Results
Source: JAMA Netw Open. 2025 Dec 1;8(12):e2545774. doi: 10.1001/jamanetworkopen.2025.45774 (PMC12670200; doi:10.1001/jamanetworkopen.2025.45774)
Supplement: Supplement 2. — Data Sharing Statement [file jamanetwopen-e2545774-s002.pdf]

Data Sharing Statement

Clark. Health Behavior of Adults Without Cognitive Impairment After Receiving Amyloid-β PET Results. *JAMA Netw Open*. Published December 01, 2025.
doi:10.1001/jamanetworkopen.2025.45774

Data
Data available: Yes
Data types: Deidentified participant data, Data dictionary
How to access data: Data request sent to Lindsay Clark at lrclark@medicine.wisc.edu.
When available: With publication

Supporting Documents
Document types: None

Additional Information
Who can access the data: Researchers whose proposed use of the data has been approved by the Wisconsin Registry for Alzheimer's Prevention executive committee.
Types of analyses: For a specified purpose.
Mechanisms of data availability: After approval of resource request by Wisconsin Registry for Alzheimer's Prevention executive committee and completion of signed data use agreement.
